# Effects of mannoprotein E1 in liquid diet on inflammatory response and TLR5 expression in the gut of rats infected by *Salmonella typhimurium*

**DOI:** 10.1186/1471-230X-10-58

**Published:** 2010-06-08

**Authors:** Sinforiano J Posadas, Victor Caz, Isabel Caballero, Emilio Cendejas, Immaculada Quilez, Carlota Largo, Marcos Elvira, Enrique De Miguel

**Affiliations:** 1 Experimental Surgery Department, La Paz Hospital, Paseo de la Castellana 261, 28046, Madrid, Spain; 2 Microbiology Department, La Paz Hospital, Paseo de la Castellana 261, 28046, Madrid, Spain

## Abstract

**Background:**

Mannoproteins are yeast cell wall componend, and rich in mannose. The use of foods rich in mannose as carbohydrate, could have a bioprotective effect against entrobacteria intestinal infection. Nothing is known about mannoproteins' activity in inflammatory bowel processes induced by entrobacteria.

This study investigates the effects of mannoprotein administration via a liquid diet on inflammatory response and TLR5 expression during intestinal tissue injury in a rat model of infection with *Salmonella typhimurium*.

**Methods:**

Adult Wistar male rats were divided into three groups: control, and mannoprotein E_1 _at 10 or 15%. Animals were fed with a liquid diet supplemented or not with mannoprotein E_1_. Groups were infected by intragastrical administration of *S. typhimurium*. 24 h post-inoculation samples of spleen, ileum and liver were collected for microbiological studies. Gut samples were processed to determine levels of proinflammatory cytokines (mRNA) and TLR5 (mRNA and protein) by quantitative PCR and Western-blot, and the number of proliferative and apoptotic cells determined by immunohistochemistry.

**Results:**

Ininfected levels of proinflammatory cytokines and TLR5 were higher in untreated controls than in the animals receiving mannoprotein. Proliferation was similar in both groups, whereas apoptosis was higher in controls. Curiosly, the mannoprotein effect was dose dependent.

**Conclusions:**

Mannoprotein administration in a liquid diet seems to protect intestinal tissue against *S. typhimurium *infection. This protection seems to expressed as a lower pro-inflammatory response and TLR5 downregulation in gut epithelium, as well as by an inhibition of apoptosis. Nevertheless, the molecular mechanism by which mannoprotein is able to regulate these responses remain unclear. These results could open up new avenues in the use of mannoproteins as prebiotics in the therapeutic strategy for treatment of inflammatory gut processes induced by microbia.

## Background

GRAM-NEGATIVE BACTERIA of the *Salmonella enteriditis *group are common human pathogens and often isolated from cases of acute food-borne gastroenteritis in developing countries as well as the United States and Europe [1]. S. enteriditis interaction with the intestinal epithelia triggers secretion of chemokines and cytokines and the subsequent luminal translocation of neutrophils [2]. The result of this inflammatory response is clinically correlated with acute diarrhea.

Intestinal epithelium is considered an integral and essential component of the innate mucosal immune system [3]. Intestinal epithelial cells (IECs) can respond to enteric pathogens (e.g. Salmonella species, Yersinia enterocolitica, and enteropathogenic Escherichia coli) either by the release of molecules directly endowed with bactericidal properties [4] or by the secretion of pro-inflammatory mediators [5-8] and the expression of adhesion molecules [9], which permit the recruitment of immune cells and induction of a protective inflammatory response that can eradicate pathogens. Many studies have demonstrated that the response by mammalian cells to pathogens is orchestrated through the activation of the nuclear transcription factor κB (NF-κB) [10,11] following cell receptor recognition of specific prokaryote motifs called PAMPs (pathogenic associated molecular patterns). Toll-like receptors (TLRs), some of which are expressed by enterocytes, are the best-characterized family of mammalian PAMPs receptors [12]. TLRs recognize an array of prokaryote motifs, including unmethylated CpG DNA motifs, lipopolysaccharides (LPS), lipoproteins, peptidoglycan, and flagellin [12,14], that are shared by both pathogenic and commensal bacteria, suggesting that either type of bacteria may have the potential to initiate innate immune host responses in IECs. Flagellin is a bacterial product that is generally considered a PAMP, with TLR5 as its physiological receptor in vertebrates [15]. In *S. typhimurium*, bacterial motilility depends on an extracellular filament structure with 20,000 subunits. Purified flagellin can activate transcription and secretion of the proinflammatory chemokine IL-8 in cell culture systems [16]. Flagellin is also a potent activator of systemic inflammation in murine models [17], and, in humans, serum levels of this protein correlate with clinical severity in bacteremic shock syndromes [18], and this indicates a role for this bacterial protein in the immunopathogenesis of inflammatory bowel disease [19]. Interestingly, recent studies indicate that flagellin is able to activate apoptotic signaling pathways. This activation is parallel to a classical proinflammatory pathway and may be a general feature of innate immunity activators of so flagellin may play a previously underappreciated role in host monitoring of, and response to, microbes [20].

Mannoproteins are components of the yeast cell wall; they are 90% carbohydrate and phosphodiester bonds between lateral manose residue and are widely used for improving the foaming properties in sparkling wines [21]. Mannoproteins are mainly N- and O- glycosilate proteins with a highly polymerized and branched glucosilade fraction, with around 150 and 200 mannose residues [22]. It has recentlybeen demonstrated that food supplementation with mannoprotein inhibits gut colonization by Salmonella and other gut bacteria in animals [23-25]. The use of foods rich in manose as carbohydrate in the diet could have a bioprotective effect against intestinal infection caused by entrobacteria [26-28]. Nevertheless, little is known about its iinvolvement in bowel inflammatory processes induced by microbes. Therefore, several clinical studies have been conducted with *Saccharomyces boulardii*, a yeast species, in the treatment and prevention of various forms of diarrhea, proving it a promising research perspective in the therapy of inflammatory bowel disease [29].

In this manuscript we evaluate the action of a yeast cell wall fraction called "mannoprotein" added to a liquid diet in a rat model of salmonella infection by analyzing the induction of proinflammatory cytokines, and, whether mannoprotein can affect hostexpression of the flagelin receptor TLR5, which is induced by Salmonella.

## Methods

### Animals

82 adult Wistar male rats were included in this study and divided in three groups depending on diets: controls (n = 24) and mannoprotein enriched (n = 58) divided by mannoprotein level (n = 28 at 10% and n = 30 at 15%). Healthy animals came from (Harlan S.A, Spain). and were housed in the Experimental Surgery facilities of La Paz Hospital, in compliance withSpanish law on the protection of experimental animals (RD 1201/2005). All procedures were approved by HULP Animal Care Committee.

### Diets

Animals were caged individually throughout the experiment with free access to. liquid diet (Dietgriff^® ^; 1 Kcal/ml, 16% Protein, 29% fat, Lipid and 55% Glucides) was supplemented either with 10% and 15% mannoprotein E_1 _(BIOMOS Alltech, Inc), mannoprotein a rich product from *Saccharomyces cerevisiae*, the resulting product was isocaloric, prepared daily and administered during 4 days. Previously different percentages (2%, 5%, 10%, 15%) of mannoprotein has been tested to identify the best percentages at which the mannoprotein injected against Salmonella colonization.

### Intragastric in vivo inoculation

Salmonella tiphymurium (ATCC 14028) were grown statically to 3 × 10^8 ^ufc/ml ( Macfarlan) and sequentially diluted to 10^3 ^ufc/ml. This concentration was the minimun needed to produce infection in the animals and to find differences between controls and mannoprotein treated animals using 10% a 15% mannoprotein diet. After four days mannoprotein administration rats were anesthetized with isofluorane (2%) and innoculated with 1ml (10^3 ^ufc/ml) of the salmonella culture in bicarbonated serum saline (30 gr/l) by intragastric catheter [30-32].

### Tissue Sampling

24 hours after innoculation, animals were killed by sodium phentobarbital i. p. administration (50 mg/Kg). Spleen, liver and ileum were collected for microbiological studies [30-32]. Samples were homogenized in sodium saline and/placed/in an Agar - Salmonella Shigella and Selenitum enriched medium. Colony identification was made its morphological characteristics and agglutination with specific anti-sera to Salmonella poly A and S. typhimurium (Difco).

Samples from the ileum, jejunum and colon were taken and placed in 10% neutral buffered formalin during 24 hr for Q-PCR and Western-blot assay. Fixed ileum was embedded in paraffin blocks, from which 5-μm serial sections were cut for immunohistochemical studies.

#### Quantification of TLR-5, IL-1β, IL-6 and TNF-α mRNA expression. Real time PCR

Expression levels of TLR-5, IL-1β, IL-6 and TNF-α were detected by real time PCR (LightCycler, Roche Diagnostics, Indianapolis, USA). cDNA from 1 μg of total RNA was used for PCR. Real-time PCR was performed with the Fast-Start DNA master SYBR Green system (Roche). The sequences of the primers are as follows:

S26-f: 5' ATGCGTGCCCAAGGACAAGG 3',

S26-r: 5'GGCAGCACCCGCAGGTCTAA 3';

IL-1beta-f: 5'AACAGCAATGGTCGGGACAT 3',

IL-1beta-r: 5'GCATTAGGAATAGTGCAGCCATCT 3';

IL-6 -f: 5'TACCCCAACTTCCAATGCTC3'

IL-6-r: 5' TGGACATTCCTCACTGTGGT 3';

TNF-α-f: 5'CACGTCGTAGCAAACC3'

TNF-α-r: 5'GGTGAGGAGCACATAGT3';

TLR-5-f:5' TGTCAACAGGGTGCTTTGTC 3'

TLR-5-r: 5'AAAAGCAGGTGGCTTGAGAA 3'

All results were normalized with respect to the expression of S26. a rhibosomal protein.

The cDNA copy number for each gene of interest was determined using a seven-point standard curve. Standard curves were run with each set of samples. Correlation coefficients (r2) for standard curves were typically ≥ 0.98. The precision of target S26 from the same cDNA wasquite good, as was the day to day reproducibility. To confirm that each primer pair correctly amplified the sequence of interest, initial PCR products from tissue were run on agarose gel, stained with 0.5 μg/ml ethidium bromide, and viewed by UV transillumination to confirm that a single product of the predicted size was produced. To confirm the specificity of the reaction product during each run, the melting profile of each sample was analyzed using the LightCycler. The melting profile was determined by holding the reaction at 80°C for 10 s and then slowly heating to 95°C with a linear rate of 0.1°C/s while measuring the emitted fluorescence. Melting curve analysis demonstrated that each of the primer pairs described amplified a single product. The rare samples that demonstrated a significant second peak in the melting profile were not used in analysis.

#### TLR-5 Western-Blot

To demonstrate potential changes in TLR5 expression, western blotting was performed on total protein samples isolated from jejune, ileon and colon tissues using RNA-DNA-protein separation reagent (Progen Industries, Brisbane, Australia). First, the protein concentration was quantified using Bradford reagent (Sigma, St. Louis MO) with BSA as a standard. Equal amounts of protein (10 μg) from each sample, and 2 μg of biotinylated broad range protein molecular weight markers (Bio-Rad, Hercules, CA) were treated with a reducing sample buffer; samples were then separated on a 12.5% SDS-PAGE minigel, and electroblotted onto 0.2-μm nitrocellulose membranes (Amersham) (100 V, 500 mA, 4°C, 40 min). Membranes were blocked for 2h in 5% bovine serum albumin (BSA) and then incubated overnight with a rabbit TLR5 polyclonal IgG antibody (Santa Cruz Inc., USA) at a concentration of 1:750. After primary antibody incubation, membranes were washed 5x10 min in tris (hydroxymethyl) aminomethane-buffered saline (TBS), with the third 10-min washing in TBS with 0.05% Tween-20, and then incubated for 1 h at 4°C with a 1:10.000 dilution of peroxidase-conjugated swine anti-rabbit biotinylated IgG and avidin/biotinylated horseradish peroxidase reagents (Dako). Membranes were then washed for 5x10 min in TBS, followed by a 60-s wash in enhanced chemiluminescence reagents (Amersham). High-performance luminescence detection film (ECL-PLUS, Amersham, Sweeden) was exposed to membranes in a film cassette for between 10s and 2 min and developed. Western-blot of β-tubuline was used as an internal control.

### Apoptosis and proliferation assay: Immunostaining

To analyze any potential site-specific changes in the member of either apoptotic or proliferative positive cells in the Ileum samples in the two groups examined, immunohistochemical detection of Ki-67 antigen or TUNEL were performed on paraffin sections of the ileum. The ki-67 was detected as follows: After removal of paraffin with xylene, by endogenous peroxidase blocking in all tissues with 3% H2O2 in methanol. All tissue sections were pretreated in a microwave oven in citrate buffer at pH 6.0 for 10 minutes at 300 W. Unspecific binding sites were blocked using washing buffer 1 (no. K5006; DAKO, Glostrup, Denmark) before incubation with the primary antibody. Slides were incubated for 1 hour at room temperature with the primary antibodies. As primary antibodies, we used monoclonal antibody MIB-5 (antibody concentration 0.2 mg/ml, no. 2093; Immunotech, Marseille, France. To avoid background due to secondary antibody binding to mouse immunoglobulins in the tissue, MIB-5 was biotinylated using the Animal Research Kit Peroxidase (no. K3954; DAKO) according to the manufacturer's instructions before immunostaining. As a detection system, a streptavidin-peroxidase complex included in the kit was used with a 3.3'-diaminobecidine (DAB) substrate (Sigma). A normal rabbit serum was used as a negative control. For TUNEL: Five-μm sections of formalin-fixed, paraffin-embedded renal tissue were dewaxed, rehydrated, washed in PBS, and incubated in terminal deoxynucleotidyl transferase (TdT) buffer for 10 min at room temperature before incubation with TdT enzyme (Promega, Madison, WI) and digoxigenin-labeled dUTP (Boehringer Mannheim, Mannheim, Germany) for 1 h at 37°C. After the sections were washed in PBS, a sheep anti-digoxigenin F(ab)_2 _was applied for 1 h at room temperature. Endogenous peroxidase was blocked, and a peroxidase-conjugated rabbit anti-sheep antibody was applied followed by a peroxidase-conjugated swine anti-rabbit antibody. The reaction was developed with the use of a Pierce metal-enhanced diaminobenzidine substrate (Rockford, IL). On the basis of terminal deoxynucleotidyl transferase mediated dUTP nick end labeling (TUNEL) staining and morphologic changes, the apoptotic cells were counted under light microscope in 50 glomerular cross sections and 10 random areas of 0.15 mm^2 ^in the interstitium, with the use of a grid at 400x magnification. Negative controls without TdT enzyme and positive controls with DNase treatment were included for each tissue. No paraffin-embedded tissues of the PAN model were available to assess apoptosis by TUNEL technique at week 17.

### Statistical analysis

Results of degree of infection, Q-PCR (cytokine and TLR5 gene expression), Western-blot (TLR5 protein levels) and immunohistochemistry (number of ki-67 antigen and apoptotic positive cells) in the different groups were compared with one-way ANOVA.

## Results

### Results of microbiological study

24 hours after Salmonella inoculation (10^3 ^ucf/ml), 9/24 (37.5%) of the animals in the control group were infected in at least one of the tissues analyzed in the microbiological analysis. Nevertheless, in the animals treated with 10% mannoprotein E_1 _the infection rate was lower 8/28 (28.6%), moreover the animals treated with 15% mannoprotein E_1 _showed an even lower infection rate --3/30 (10%)-- and only one tissue type was infected in 11 of the 58 mannoprotein-treated animals. Interestingly, the Salmonella dosis was optimized to 10^3 ^ucf/ml, the level at which there were differences between groups treated with mannoprotein. The first comparison of infection levels between two groups (controls and 10% mannoprotein E_1_) was statistically non significant with a p < 0.54 and a difference of 8.9%, meaning that although mannoprotein had reduced infection, the decrease was not significant. Comparing controls with rats receiving 15% mannoprotein E_1 _showed significant differences in infection rate (p < 0.05), as the percedntage of infected animals (intestinal colonization) was reduced by 27.5%, thus indicating a dose dependency (difference control vs 10% vs 15%). Interestingly, infection was found in one or more tissues in the control group animals, whereas in the mannoprotein groups, no animal had infection in more than one tissues type, and in all cases the infection was limited to the ileum homogenates, which necessarily included material from the lumen. Notably, when we evaluated bacterial translocation (regional and/or systemic levels), mannoprotein resulted in a significant decrease from intestinal lumen (local level) to liver and spleen since 44% of controls showed translocation, and 0% of either of the 10% and 15%. mannoprotein treated groups showed infection beyond the local level.

Table 1 shows the results in percentages of the degree of infection and number of tissues affected in all study groups.

**Table 1:** Results of microbiological study

Group	N	% infected Rats	% of infected rats with ≥ 2 organs affected	% of infected rats with 1 organ affected
Control	24	37.5 (N = 9)	44.4% of 37.5% (N = 4)	55.6% of 37.5% (N = 5)

Mannoprotein 10% E1	28	28.6 (N = 8)	0%	100%(N = 8)

Mannoprotein 15% E1	30	10% (N = 3)	0%	100% (N = 3)

Tissue was taken from the Salmonella infected rats for molecular biology and immunohistochemistry study (control, n = 9; mannoprotein 10% E1, n = 8). The number of infected rats (n = 3, 10%) in the Manoprotein 15% E1 group was too low to allow statistically significant analysis and so were not included in the following studies.

### Analysis of mRNA expression of IL-6, TNF-alpha, IL-1beta and TLR5 in ileal, colon and jejunum samples

Proinflammatory cytokines and TLR5 were determined by quantitative RT-PCR analysis, which measured the changes in gene transcription. Changes were significant at the mRNA level in intestinal ileum, colon and jejunum mucosa.,.

#### mRNA expression in jejunum

**Figure 1:**
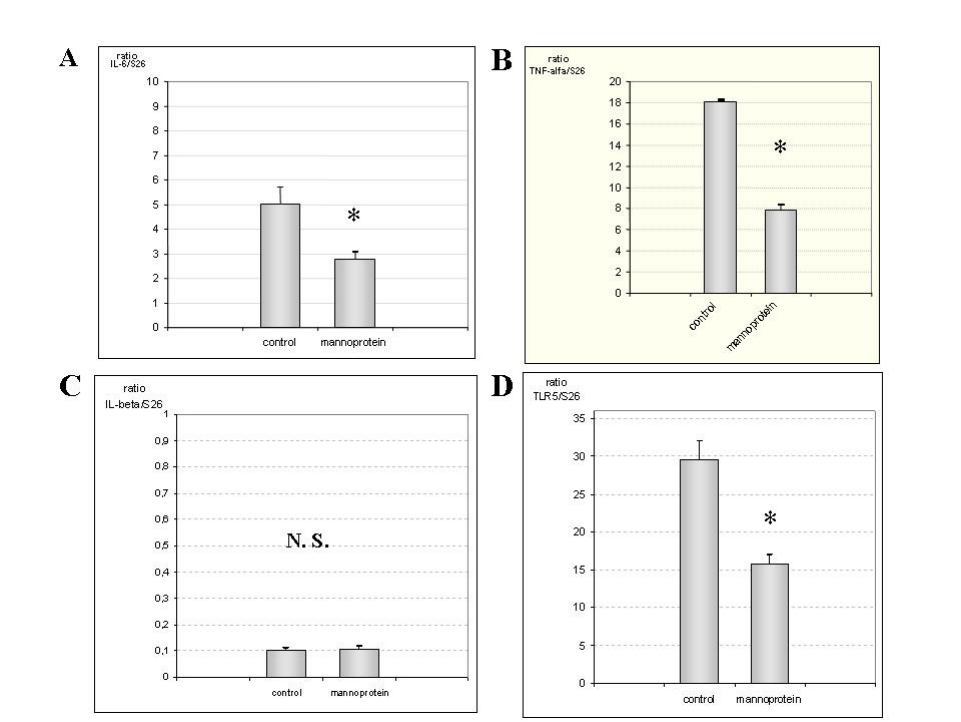
**Quantification of IL-6, TNF-α, IL-1β and TLR5 mRNA in jejune samples**. Figure 1A and 1B illustrate in a bar diagrams that IL-6 and TNF-α mRNA expression increased markedly in control group compared with mannoprotein. Figure 1C shows IL-1β mRNA expression in both groups represented as bars diagram (n = 9 control group, n = 8 mannoprotein group). No statistical differences were observed between both groups. Finally, figure 1D shows the results for TLR5 gene expression in both groups with augmented levels in the control group compared with mannoprotein. Error bars represent the standard deviations. * Significant at p > 0.05 compared with control.

Figures 1A,B,C and 1D illustrate the results for IL-6, TNF-alpha, IL-1beta and TLR5 levels respectively as a ratio of mRNA expression compared with the expression of the S26 gene, which was employed as a housekeeping gene. IL-6 levels were higher in the control group than in the mannoprotein-treated animals (p = 0.001; figure 1A). Levels of TNF-alpha and IL-1beta were also higher in the control group than the mannoprotein treated animal group (p = 0.001) in both analyses. Levels of TLR5 mRNA expression also differed significantly between groups (p < 0.001, figure 1D).

#### mRNA expression in Ileum

**Figure 2:**
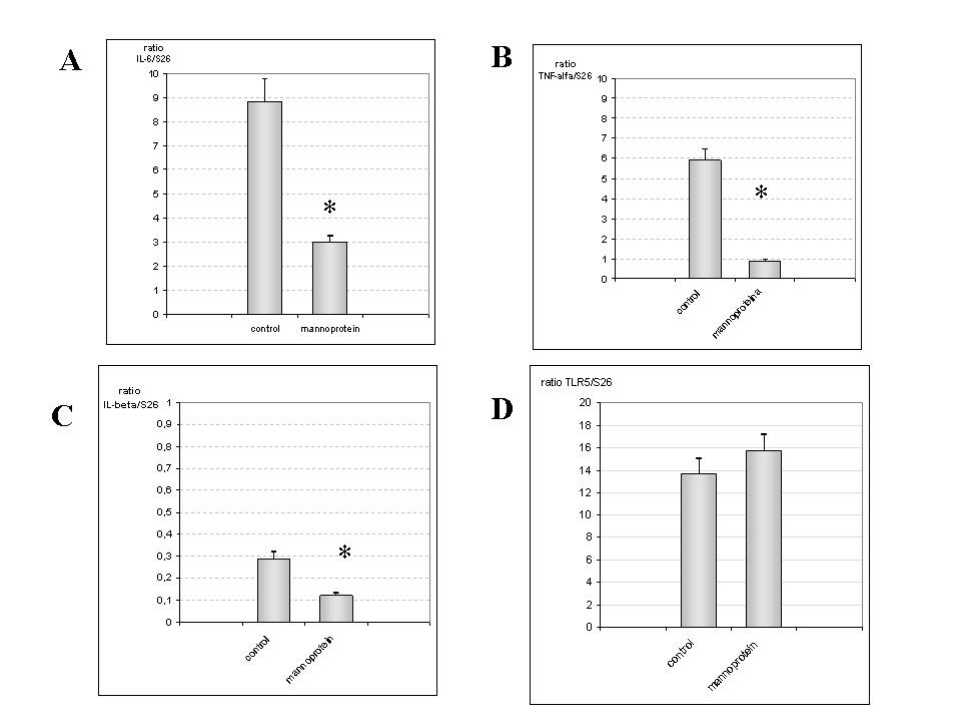
**Quantification of IL-6, TNF-α, IL-1β and TLR5 mRNA in Ileum samples**. Figure 2A and 2B illustrate in a bar diagram that IL-6 and TNF-α mRNA expression increased markedly in control group compared with mannoprotein(n = 9 control group, n = 8 mannoprotein group). Figure 2C shows IL-1β mRNA expression in both groups represented as a bar diagram, with higher and significant levels in the controls. Finally, figure 2D shows the results for TLR5 gene expression in both groups with no differences in the control group compared with mannoprotein. Error bars represent the standard deviations. * Significant at p < 0.05 compared with control.

As in the jejunum, IL-6, TNF-alpha and IL-1beta levels were also higher in the control group than in the mannoprotein treated group (p < 0.001) (figures 2A,B and 2C). However, TLR5 mRNA expression levels showed no differences between the two groups (figure 2D).

#### mRNA expression in Colon

**Figure 3:**
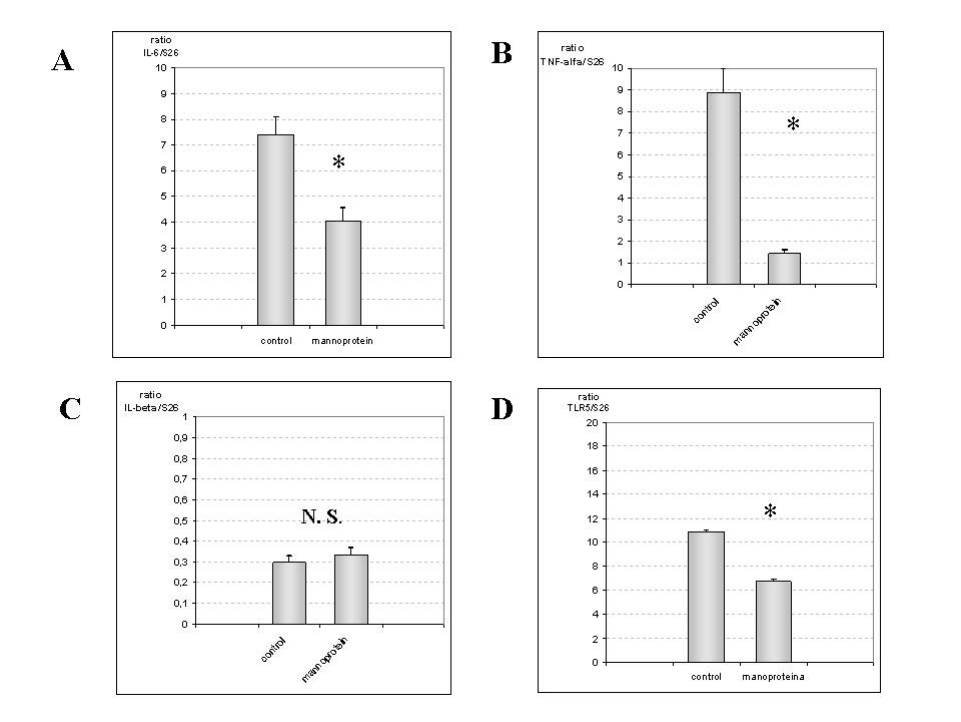
**Quantification of IL-6, TNF-α, IL-1β and TLR5 mRNA in colon samples**. Figure 3A and 3B illustrate in a bar diagram that IL-6 and TNF-α mRNA expression increased markedly in control group compared with mannoprotein (n = 9 control group, n = 8 mannoprotein group). Figure 3C shows IL-1β mRNA expression in both groups represented as a bar diagram. No statistical differences were observed between both groups. Finally figure 3D shows the results for TLR5 gene expression in both groups with augmented levels in the control group compared with mannoprotein. Error bars represent the standard deviations. * Significant at p < 0.05 compared with control.

Proinflammatory cytokine mRNA expression and TLR5 levels were similar in colon to levels in jejunum with the controls showing higher IL-6 levels than the mannopprotein-treated animals (p < 0.001; figure 3A). TNF-alpha and IL-1beta levels were higher in the control group than the mannoprotein-treated group (p < 0.001) in both analyses (figure 3B and 3C). TLR5 gene transcription levels also showed statistical differences between the two groups. p < 0.001; figure 3D.

#### Protein levels of TLR5 in jejunum, Ileum and Colon

**Figure 4:**
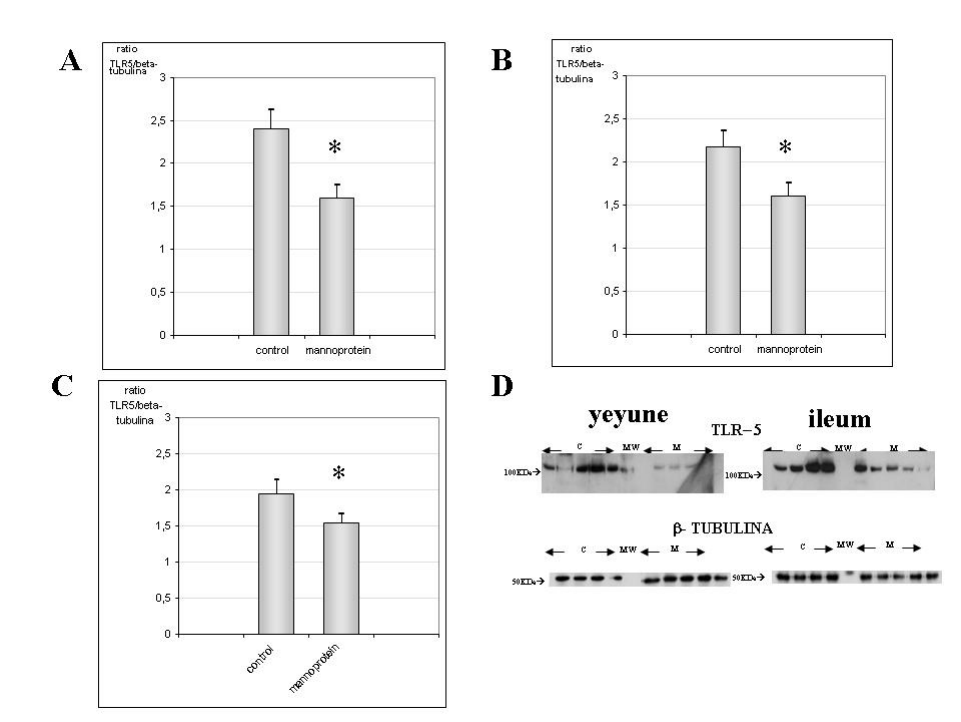
**Quantification of TLR5 protein levels in yeyune, Ileum and colon samples**., Figure 4A, 4B and 4C show the levels of TLR5 protein expression in both groups for yeyune, ileum and colon respectively. In all figures an augmented expression of TLR5 is observed in the control group compared with mannoprotein (n = 9 control group, n = 8 mannoprotein group). These differences were statistically significant. Figure 4D shows a representative Western-blot picture of TLR5 and β-tubuline in yeyune (left) and Ileum (right) for both groups controls (designed as C) and mannoprotein (designed as M). Error bars represent the standard deviations. * Significant at p < 0.05 compared with control.

The results of TLR5 protein expression showed higher levels in the control group than the mannoprotein-treated group in the three tissues studied (figure 4). TLR5 levels were similar in jejunum, Ileum and colon in the controls. The greatest significant differences between groups were found in the Ileum (p < 0.009, figure 4B). However, the differences in TLR5 expression levels in jejunum and colon seem to be similar in all groups (p < 0.05, figure 4A and 4C). Figure 4D illustrates a representative picture of TLR5 and beta-tubuline Western-Blot result for both groups in jejunum and Ileum.

## Immunohistochemical findings

### Results on proliferation and apoptosis

Apical Ki-67 antigen immunohistochemistry in the intestinal Ileum and colon crypts and villi

**Figure 5:**
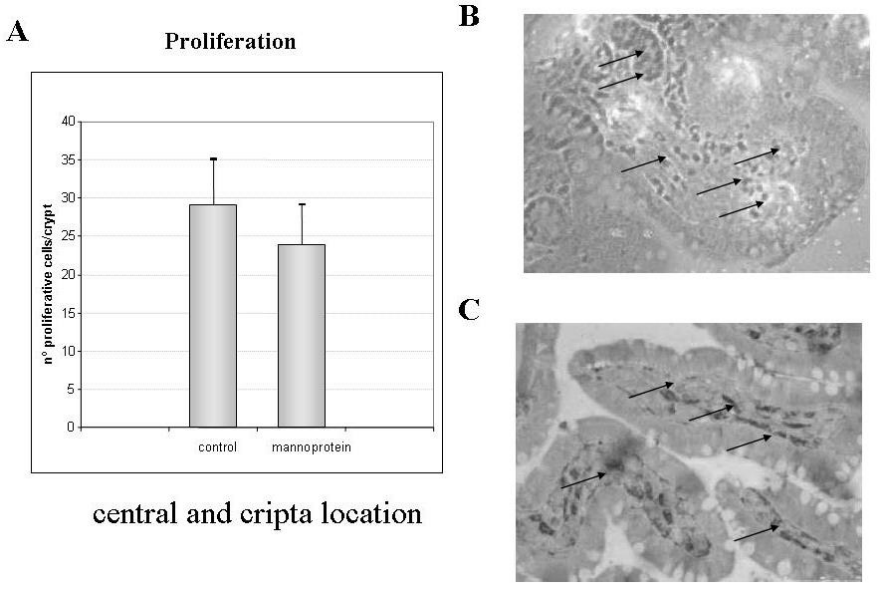
**Number of total proliferative immunoreactive cells in ileum crypts and villi**. Figure 5A shows the number of total proliferative positive cells in both groups analyzed as a bar diagram. Non statistical differences were found between controls and mannoprotein group. Figure 5B shows immunohistochemical location of proliferative cells in Ileum crypt in control. Figure 5C shows immunohistochemical location of proliferative cells in crypt villi in mannoprotein. Arrows indicate proliferative positive cells predominantly located in central villi and crypta. Photomicrographs at original magnification 20x. Error bars represent the standard deviations. * Significant at p < 0.05 compared with control group.

The data showed insignificant differences in the number of Ki-67 positive cells in the different study groups. Positive cell staining for proliferation was found mainly in the central villi and crypts. The bar diagram in figure 5 A illustrates the results in number of positive cells per villi for both the control and the mannoprotein treated groups. Figure 5B shows a representative picture of tissue and positive staining (arrows) for each group.

**Figure 6:**
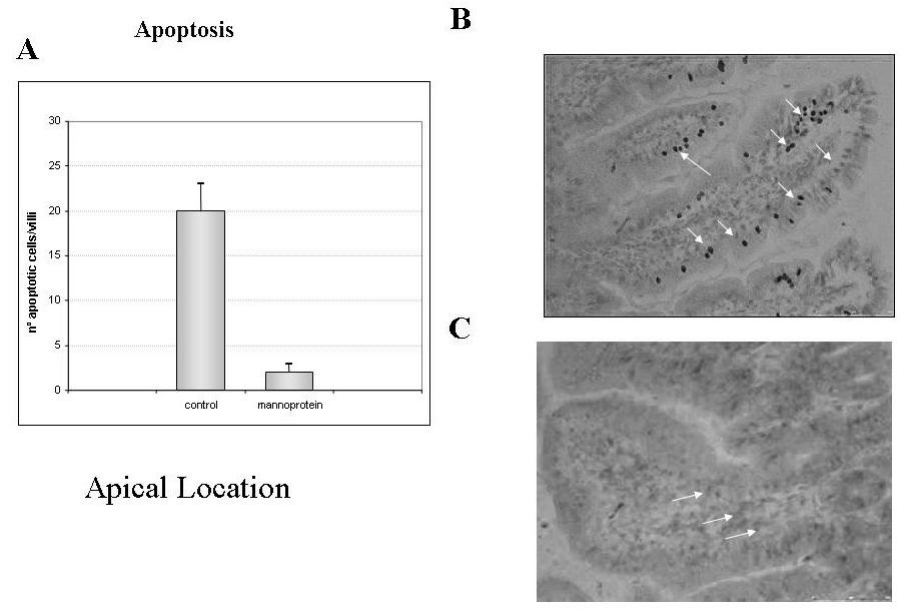
**Number of total apoptotic immunoreactive cells in ileum villi**. Figure 6A shows the number of total apoptotic positive cells in both groups analyzed as a bar diagram. Significant statistical differences were found between controls and mannoprotein group. Figure 6B shows immunohistochemical location of apoptotic cells in Ileum villi in control. Figure 6C shows immunohistochemical location of apoptotic cells in Ileum villi in mannoprotein. Arrows indicate proliferative positive cells predominantly located in apical. Photomomicrographs in original magnification 20x. Error bars represent the standard deviations. * Significant at p < 0.05 compared with control group.

Regarding apoptosis immunoreactivity, our data showed significant differences between the two groups (p < 0.001). Interestingly, apical immunoreactiviy showed an increase in the control group compared to the mannoprotein treated group - see figure 6A. These results are expressed in a bar diagram indicating number of positive cells per villus. Figure 6B shows a representative picture of tissue and positive staining (arrows) for each group.

## Discussion

Recent molecular-based investigations have confirmed the species diversity and metabolic complexity of human microbiota. It is also increasingly clear that human gut microbiota have a crucial impact on host health, both as a source of infection and environmental insult and, conversely, as protection against disease by maintaining gut function. Thus, investigators are beginning to develop microbiota management employing by probiotics and prebiotics, including mannoprotein [33]. Nevertheless, little information is available in the literature to analyze the effects in the gut of the molecular components of pro-prebiotics during a bacterial infection. This manuscript evaluates the effects of mannoprotein, a component of the yeast cell wall, in a rat model of Salmonella infection. Microbiological analysis revealed a protective effect by these compounds that decreased the number of animals infected and the number of organs (spleen, liver and gut) invaded by the infection. Furthermore, at a molecular level, the infected animals treated with mannoprotein showed a lower pro-inflammatory response than the untreated infected control animals, which did not receive mannoprotein.

It is generally assumed that an intestinal proinflammatory mechanism is a characteristic response to pathogenic intestinal bacteria, whereas the commensal bacteria should not induce this response, which interrupt the symbiosis between the bacteria and their mammalian host. Epithelial cells lining the gut have recently been identified as key players in the regulation of the initial steps of host proinflammatory responses to intraluminal bacteria via their controlled expression of PAMP receptors. One attractive hypothesis is that intestinal epithelial cells are hyporesponsive to commensal intraluminal bacteria because of the low PAMP receptor number on the apical surface on the epithelial cells [22], whereas invasive bacteria have access to, and can activate, PAMP receptors in the intracellular space or on the basolateral membrane. In support of the hypothesis, our study shows that mannoproteins apparently modulate the proinflammatory response either because they form a physical barrier to the Salmonella infection or they disrupt the interaction required for activation of NF-κB at a given molecular level, thus blocking further signalling cascades beyond that level. Induction of the NF-κB pathway relies on the interaction of flagellin, which is common to all flagellated bacteria, with the TLR5 expressed *in situ *in the ileum, in both the basal and apical compartments of the epithelial cells and enterocytes. In fact, mannoproteins inhibited campylobacter adherence and invasion of Caco-2 cells in a recent in vitro study [34]. Thus, mannoproteins would seem good candidates to protect against infection through an intestinal route.

In our study, an inflammatory cytokine response was induced in the gut of the rats after oral infection with small amounts of a S*almonella typhimurium innoculus*. In our experiments the control group showed higher TNF-α and IL-6 mRNA expression than the mannoprotein-treated rats. This phenomenon was observed in the three tissues analyzed: jejunum, ileum and colon. IL-1β mRNA expression was only affected in the Ileum of control rats, which showed higher expression levels than treated animals. This response agrees with previous studies in murine small intestine, in which both IL-6 and TNF-α were induced after exposure to *S. typhimurium *[35-38]. IL-1β mRNA synthesis is increased in intestinal epithelial cells after *Salmonella *infection [36]. In contrast to the other cytokines studied, IL-1β enhances the innate response by activating NF-κB [39,40]. We observed a clear action by mannoprotein on proinflammatory cytokine mRNA expression since these levels were lower in the treated animals than the controls. This suggests an anti-inflammatory effect whereby mannoprotein could be involved in some protective mechanisms against oral infection by *Samonella typhimurium*.

Our study also measured the expression of TLR5, the specific *Salmonella typhimurium *flagellin receptor, at both mRNA and protein levels. Our results demonstrated decreased levels of gene and protein expression in the mannoprotein treated group. Thus, mannoprotein itself is able to regulate TLR5 expression after infection by *Salmonella*. No data are available in the literature about this regulation, but there are recent studies on TLR5/flagellin interaction and the activation cascades involved in this process. These signaling cascades activate the NF-κB pathway and induce a proinflammatory response that mediates up-regulation of cytokines, chemokines and adhesion molecules, etc. Protein modifiers of cellular apoptotic pathways are integral components of this response. This observation strongly supports the notion that apoptotic activation proceeds in parallel with proinflammatory activation [41-43]. Concerning the inflammatory response in our results, the decreased TLR5 levels of in the mannoprotein group could explain the lower levels of IL-1β, TNF-α and IL-6 mRNA expression found in treated rats compared to their controls.

The crypt proliferative cells and apical and central apoptotic cells of the villi were immunohistochemically analysed in order to establish a possible effect of mannoprotein in proliferative and apoptotic response in Ileal tissue. Interestingly, the number of apoptotic cells in the control group was significantly higher than in the mannoprotein group. Nevertheless, when we analyzed the proliferative response in tissue these were no differences between the groups. This could be explained by the fact that the highest levels of TLR5 were found in the control group where they possibly activated both the pro-inflammatory and the apoptotic pathways as suggested in a recent study where both pathways were activated the inappropriate location of flagellin [25]. We cannot confirm this possibility, although it seems likely, because we did not determine flagellin levels or position in our study. Furthermore, other in vitro studies have indicated that Salmonella provokes apoptosis in macrophages [44,45] and in HT-29 epithelial cells after 24 hours of co-culture [46]. On a different level a recent study with chickens, reports that mannoprotein diet enrichment increased jejunal villus height and thymal weight, changes that could point to an increased immune response capacity and improved gut function [47]. Summarising, the administration of mannoprotein could exert a protective effect by inhibiting the apoptosis induced by oral infection with *Salmonella*.

In conclusion, Mannoprotein administration in a liquid diet seems to protect the intestinal tissue against the effects of *Salmonella triphymorium*. This protection seems to be effected by down regulation of the proinflammatory response and of TLR5 expression, in addition to the inhibition of apoptosis. Nevertheless, the molecular mechanism by which mannoprotein is able to regulate this response remains unclear and is still under investigation. These results could open up new avenues in the therapeutic strategy for treatment of inflammatory gut processes induced by microbia.

## Conclusions

Mannoprotein administration in a liquid diet seems to protect intestinal tissue against *S. typhimurium *infection. This protection seems to expressed as a lower pro-inflammatory response and TLR5 downregulation in gut epithelium, as well as by an inhibition of apoptosis. Nevertheless, the molecular mechanism by which mannoprotein is able to regulate these responses remain unclear. These results could open up new avenues in the use of mannoproteins as prebiotics in the therapeutic strategy for treatment of inflammatory gut processes induced by microbia.

## Competing interests

The authors declare that they have no competing interests.

## Authors' contributions

The work presented here was carried out in collaboration between all authors. SJP and EM defined the research theme. SJP, CL and EM designed methods and experiments, carried out the laboratory experiments, analyzed the data, interpreted the results and SJP and EM wrote the paper. VC, IQ, ME, EC, CL and IC co-designed the experimental model, quantitative PCR, Western-blot and Immunohistochemistry experiments, and co-worked on associated data collection and their interpretation. All authors have contributed to, seen and approved the manuscript.

## Pre-publication history

The pre-publication history for this paper can be accessed here:

http://www.biomedcentral.com/1471-230X/10/58/prepub
